# Informal over-the-counter supply of antibiotics in Ghana: A qualitative analysis of practices in community pharmacies

**DOI:** 10.1016/j.rcsop.2025.100696

**Published:** 2025-12-10

**Authors:** Radolf Ansbert Nortey, Irene Akwo Kretchy, Mercy Naa Aduele Opare-Addo

**Affiliations:** a Department of Pharmacy Practice, Faculty of Pharmacy and Pharmaceutical Sciences, Private Mail Bag, University Post Office, Kwame Nkrumah University of Science and Technology, Kumasi, Ghana; b Department of Pharmacy Practice and Clinical Pharmacy, School of Pharmacy, University of Ghana, P.O. Box LG 43, Legon, Accra, Ghana

**Keywords:** Antibiotics, Regulations, Supply, Pharmacy, Policy, Resistance, Ghana

## Abstract

**Background:**

Antibiotics are among the most widely prescribed medicines and fall within a well-defined framework for access and supply. Despite existing regulatory systems for antibiotic control, weak regulatory enforcement has led to non-prescription access from community drug retail outlets and widespread self-medication with antibiotics.

**Objective:**

To explore the factors associated with the over-the-counter supply of antibiotics within community pharmacies across Ghana.

**Method:**

The exploratory qualitative study employed semi-structured interviews. The study population consisted of pharmacy practitioners randomly recruited from the medicine retail outlets situated in rural, peri-urban, and urban communities in Ghana. The interview questions were organised within the framework of the theory of planned behaviour and investigated participants' attitudes, social norms, and perceived control over antibiotic use. The data was transcribed, coded, and thematically analysed using NVivo version 10.

**Results:**

Twenty-three pharmacy practitioners (i.e., pharmacists, pharmacy technicians, and medicine counter assistants) participated in the study. Participants described how economic incentives, sales targets, and the perceived social status of customers often pressured them to supply antibiotics without prescriptions, especially in an environment where regulatory oversight was viewed as weak or absent. Many noted that customers felt increasingly entitled to request antibiotics, drawing confidence from online health information and limited public education. Although the participants knew about antibiotics and antimicrobial resistance, their awareness of existing national antimicrobial policies was limited.

**Conclusion:**

The over-the-counter supply of antibiotics in Ghana is fuelled by various factors that differ slightly along the lines of community urbanisation and development. Policy makers must take full cognisance of these factors and adopt community-tailored strategies that target medicine retailers.

## Highlights

• Non-prescription supply of antibiotics is influenced by various socio-economic factors
• It is more likely to meet pharmacists in pharmacies within urban areas than pharmacies in rural zones.
• Pharmacy practitioners are unaware about existing antimicrobial policies.
• Policy actors need to develop a guidance framework for pharmacists to streamline the supply of antibiotics.

## 1 Background

Antibiotics are generally classified as prescription medicines and fall within a well-defined framework for access and supply.^1^ Around the world, antibiotics remain among the most frequently prescribed medications.^2^ The increasing rate of antibiotic consumption has been attributed to multiple factors, including economic growth and an unwarranted ease of access.^3^ This situation has been linked to the increasing rate of antimicrobial resistance (AMR), which is a major public health concern worldwide.^4^, ^5^, ^6^

To effectively combat antimicrobial resistance, it is essential to address both global and industry-wide strategies as well as the specific practices contributing to the problem. Although the emergence of AMR is multifactorial and a natural phenomenon, the misuse and overuse of antibiotics play significant roles in accelerating this process of resistance.^7^ This inappropriate use of antibiotics is typically associated with non-prescription access to or supply of antibiotics from community drug retail outlets.^2^^,^^8^^,^^9^

Community drug retail outlets are core to the global pharmaceutical supply chain system and often include pharmacies, over-the-counter medicine sellers (OTCMS), and accredited drug dispensing outlets.^8^^,^^10^^,^^11^ The general proliferation of antibiotics in these outlets amidst weak regulations, especially in developing countries, is of grave concern and creates a state of emergency that threatens global health security.^9^^,^^12^^,^^13^

In Ghana, most antibiotics are classified as prescription-only medicines under the regulatory oversight of the Food and Drugs Authority (FDA).^14^^,^^15^ Despite this well-defined legislative framework for control, antibiotics are supplied and distributed from community drug retail outlets without any regard to the existing FDA classification and permissions.^11^^,^^16^

Furthermore, the COVID-19 pandemic worsened this landscape of antibiotic control and heightened the phenomenon of self-medication around the world.^11^^,^^17^^,^^18^ Pharmacy practitioners and clinicians expressed various concerns, such as fear, anxiety, and information paucity, that served as a self-justification for the over-the-counter supply of antibiotics.^18^ Nortey et al. (2021) described this COVID-19-induced use of antibiotics as worrisome and called for a more decisive coordinated action on antimicrobial stewardship.

The non-prescription distribution and use of antibiotics in Ghana have been popularly associated with rural communities and OTCMS.^19^^,^^20^ Education, gender, income, and lax regulatory structures are common factors often associated with this growing trend of antibiotic self-medication in rural communities.^21^, ^22^, ^23^ In addition, economic incentives and over-patronage of health facilities have also been implicated as key drivers in the non-prescription supply of antibiotics from the OTCMS.^20^^,^^23^ Thus, there is some association between urbanisation and community utilisation of antibiotics.^23^ Studies on antimicrobial stewardship in Ghana tend to focus on the prevalence of OTCMS and their poor adherence to regulatory protocols.^20^ Despite the contributory role of OTMCS in irrational antibiotic use, seemingly registered and regulated pharmacies in the urban sector also play a large part in this development.^11^^,^^24^ Existing studies have primarily focused on rural settings and household or community perspectives regarding antibiotics misuse.^11^^,^^19^^,^^20^^,^^25^ However, there is limited information on dispensing behaviour among suppliers in urban settings and a lack of comparative data across other geographical zones in Ghana.^26^^,^^27^ The research draws on the Theory of Planned Behaviour (TPB) as a theoretical framework to analyse the behavioural influences shaping this practice. The TPB is often used to predict human behaviour or intentions, and has been widely used in health research.^28^ The TPB posits that behavioural intention informs the likelihood of an individual performing a behaviour and that this intention is impacted by attitudes, subjective norms, and perceived behavioural control.^29^

To address this gap, the present study explores the factors associated with the over-the-counter supply of antibiotics within community pharmacies across rural, urban, and peri-urban settings in Ghana, drawing on the Theory of Planned Behaviour (TPB) as an interpretive lens.

## 2 Method

### 2.1 Study design

An exploratory qualitative study was conducted to examine the social factors associated with the over-the-counter supply of antibiotics in Ghana. The study employed semi-structured interviews conducted between December 2022 and February 2023.

### 2.2 Study sites

This research was conducted in the Greater Accra Metropolitan Area (GAMA) because of its populous and industrial nature. The majority of community pharmacies in Ghana are situated within this region, with more facilities added each day.^11^^,^^30^ The study was carried out in three (3) specific areas located within three purposively selected districts in the GAMA: La Dade-Kotopon Municipal, Ga West Municipal District and Shai Osudoku to represent urban, peri-urban, and rural communities, respectively. The La Dade-Kotopon Municipal District is an entirely urban jurisdiction with the lowest poverty depth within the GAMA.^31^^,^^32^ The Shai Osudoku district, on the other hand, is predominantly rural (76.7 %) and has the highest level of poverty (23.2 %) in the region.^31^^,^^33^ The Ga-West Municipal District is described as a “peri-urban Accra”. It exhibits characteristics of both rural and urban settings, with a more significant population transitioning from rural to urban areas.^32^^,^^34^ Cantonments/Labone, Dodowa, and Medie were conveniently sampled from the urban La Dade-Kotopon Municipal District, rural Shai Osudoku District, and peri-urban Ga West Municipal District, respectively.

### 2.3 Study population

The study population consisted of twenty-three pharmacy practitioners purposively sampled from the medicine retail outlets in the selected communities. Purposive sampling was adopted to ensure variation across practitioner roles, including pharmacists, pharmacy interns, pharmacy technicians and medicine counter assistants. This approach enabled the inclusion of participants with diverse experiences relevant to the provision of pharmaceutical services in different community settings.

Data collection continued until no new themes or insights emerged, indicating that data saturation had been achieved. By the twenty-third interview, participants were reiterating similar views, suggesting that additional data would not yield substantially new information. Guided by the principle of saturation and the quality of the data collected, recruitment was concluded.^35^ The resulting sample size was large enough to enable the identification of novel interpretations of the phenomenon under study and small enough to ensure thorough navigation of each case.^36^^,^^37^

### 2.4 Data collection

A well-designed interview guide was developed and adopted to ensure the credibility, authenticity, and integrity of the data collected from respondents.^36^ The interview questions were structured to elicit the salient behavioural, normative, and control beliefs within the framework of the Theory of Planned Behaviour in the context of non-prescription antibiotic dispensing (Table 1). Salient beliefs are the immediate thoughts that readily come to mind when the interviewees are posed with specific open-ended questions, and the modal salient beliefs are the thoughts common to the sample population.^37^

**Table 1:** Interview guide based on the Theory of Planned Behaviour.

Construct	Item from Interviewer Guide
Attitudes & Beliefs	1. Under what circumstances would you consider providing antibiotics over the counter? 2. What are the potential benefits and risks (if any) associated with dispensing antibiotics without a prescription?
Subjective Norm	3. How do your clients react to your cooperation or disagreement to dispense antibiotics over the counter? 4. Which individuals or groups would support your decision to dispense antibiotics over the counter?
Perceived Behavioural Control	5. What circumstances or conditions make it easier to dispense antibiotics without a prescription? 6. What conditions or individuals make it difficult to dispense antibiotics over the counter?
Knowledge	7. How would you describe the role of antibiotics and the issue of antimicrobial resistance? 8. What policies or laws regulate the use of antibiotics and guide antimicrobial resistance (AMR) prevention efforts in Ghana?

The interview guide consisted of open-ended items that encouraged participants to share their experiences and beliefs regarding non-prescription antibiotic dispensing. The process of data collection enabled homogeneity in the data for easy characterisation and analysis.^38^ This interview guide was piloted with five community pharmacists in Accra, Ghana, to ensure that the questions had the inherent ability to elicit the right information. After the pilot interviews, the questions were slightly refined through a dialogic and iterative process.^39^ For instance, the question on factors facilitating non-prescription antibiotic use was revised to “What conditions or circumstances make it easier for OTC antibiotic dispensing?”. Additionally, specific questions about COVID-19 were incorporated into the follow-up questions based on feedback from the pilot group.

All interviews were conducted face-to-face in English by the first author (RAN), a male pharmacist and PhD candidate in Social Pharmacy with experience in both qualitative and quantitative research. The study participants were identified and recruited in person at selected community pharmacies. During each visit, the lead practitioner on duty was approached. Where a licensed pharmacist was present, they were invited to participate in the study. In cases where no pharmacist was available, the lead MCA or the most senior staff member on duty was considered. To ensure consistency, the interviews were conducted between 10:00 a.m. and 2:00 p.m. across all sites. Most participants agreed to be interviewed immediately following their consent. In a few instances, interviews were scheduled at a later time that was convenient for the participant. All interviews were conducted on-site within the pharmacy. In pharmacies with a private consultation room or office, interviews were conducted in those spaces with little to no interruption. However, in most cases, interviews took place in the open pharmacy area. To enhance privacy, interviews were conducted in quieter sections of the pharmacy, away from the main service counter. Despite these efforts, occasional interruptions occurred when clients required the interviewer's attention. In such cases, interviews were temporarily paused and resumed once the practitioner was available. The interviews lasted approximately 30 min, with the longest interview lasting 46 min. The sessions were recorded with a digital voice recorder.

### 2.5 Data management and analysis

The interviews were audio-recorded, transcribed verbatim, and analysed (by RAN and IAK) using thematic data analysis guided by the TPB model.^40^^,^^41^ The main thematic areas of the interviews were synthesised and categorised using a stepwise process which included familiarisation with the data, coding the data and identifying themes.^40^^,^^42^

To start, the analysts acquainted themselves with the transcripts and created a broad thematic template based on the interview questions, including open elements for concepts outside the TPB model. Subsequently, a codebook was developed to capture the main themes and subthemes. The major themes were determined based on the common patterns identified across the participants' responses.

To enhance credibility and coding reliability, RAN and IAK independently coded a subset of transcripts and compared their interpretations. Differences were discussed and resolved through peer debriefing, and the research team refined the results until they reached a consensus on the final themes/subthemes, along with supporting quotes from the interviewees. Member checking was carried out with selected participants to validate the emerging themes and confirm that the interpretations accurately reflected their experiences and perspectives.

Qualitative Research software NVivo, Version 10, was employed to facilitate the coding and data management processes.^43^ The data collection and analysis were guided by the COREQ checklist (Online Resource 1) to ensure the credibility, transferability, and originality of the study.^44^^,^^45^

### 2.6 Ethical approval

The Ghana Health Service Ethics Committee approved this study on 11 October 2021, under registration code GHS-ERC: 008/05/21. To guarantee participant confidentiality, no personal identifiable information was collected or linked to any of the recordings. In accordance with ethical protocols, all the interviewees were briefed on the purpose of the research and signed a written informed consent form before participating in the study.

## 3 Results

### 3.1 Characteristics of study participants

Out of the 23 pharmacy practitioners who took part in the study, 9 (39.13 %) were males and.

14 (60.87 %) were females. 8 (35 %) participants were between the ages of 20 and 30, 12 (52 %) were between 31 and 40, and 3 (13 %) were above 40 years. The majority of the respondents were medicine counter assistants, with 12 (52 %) and 10 (43 %) being pharmacists. The characteristics of the study participants are summarised in Table 2.

**Table 2:** Characteristics of the Study Participants.

Characteristics	Frequency	Percentage (%)
**Sex**		
Males	9	39.13
Females	14	60.87
**Age**		
20–30	8	35
31–40	12	52
> 40	3	13
**Educational Status**		
BPharm/PharmD	10	43
Higher National Diploma in Dispensing Technology	1	4
MCA Certificate	12	52
**Years of Practice**		
1–5	9	39
6–10	11	48
>10	3	13
**Status in Pharmacy**		
Locum Pharmacist	4	17.39
Superintendent Pharmacist	3	13.04
Supervisor MCA	15	65.22
Pharmacy Owner	1	4.35

### 3.2 Key themes associated with the OTC supply/demand of antibiotics in the framework of the theory of planned behaviour (TPB)

**Fig. 1:**
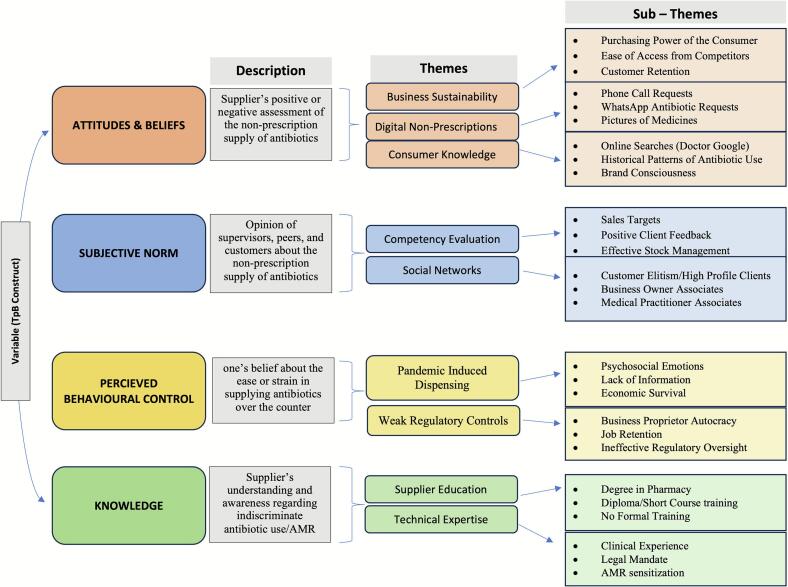
Factors associated with the OTC supply/demand of antibiotics based on the TPB.

The interview transcripts provided information within the framework of the TPB model. The main variables of the model (Attitudes/Beliefs, Subjective Norms, Perceived Behavioural Control and Knowledge) served as the major domains for the emerging themes and corresponding sub-themes. The thematic results highlight the factors associated with a pharmacy practitioner's decision to waive the regulatory requirement for a prescription when dispensing antibiotics in community pharmacies (Fig. 1). Quotations from participants are italicised and are represented by their roles, interview number, and the geographic location of the pharmacy.

## 4 Attitudes and beliefs

### 4.1 Themes

1–3 correspond to the “Attitudes and Beliefs” construct of the TPB framework. They capture practitioners' evaluative perspectives about OTC antibiotic supply, whether viewed as a positive business strategy, a response to perceived consumer competence, or an acceptable practice when clients presented informal prescriptions.

### 4.2 Theme

1: Business Sustainability

• Purchasing Power of the Consumer

Practitioners mentioned financial gain as an incentive for the non-prescription supply of antibiotics, especially considering the purchasing power of the customer. It is common for pharmacy practitioners to profile customers and serve medicines in line with the perception of opulence.

We are only able to meet our targets when we get customers who arrive by car or bring prescriptions from Dodowa Hospital. Therefore, we don't joke with our car-holding customers. (MCA #3, Rural).

• Ease of Access from Competitors

Respondents indicated that strict enforcement of antibiotic control was counterproductive, as other pharmacies within the catchment area would readily serve the medicines over the counter. The ease of access to antibiotics from competing facilities was toxic to pharmacies that chose to enforce stricter controls.

• Customer Retention

Some pharmacy practitioners described the over-the-counter dispensing of antibiotics as a norm and a critical aspect of maintaining customer loyalty. Clients continuously patronised the facility because they knew their requests would be met with minimal resistance.

### 4.3 Theme

2: Digital Non-prescription.

• Phone Call Requests/WhatsApp and Pictures of Medicines

It was common for pharmacy clients to use digital channels, such as phone calls, text messages (SMS), social media (Snapchat, WhatsApp, etc.), and multimedia, to communicate antibiotic requests. This frequently occurred in urban and peri-urban communities. Community members in peri-urban areas frequently requested antibiotics by presenting images of the packaging. Members in urban communities often make such requests via texts received or a supposed clinician ready to dictate the intended medical prescription over a phone call.

A client comes in and passes a phone to you: “My doctor wants to talk to you”. The doctor proceeds to dictate the antibiotic prescription via phone (Pharmacist #1, Urban).

### 4.4 Theme

3: Consumer knowledge.

• Online Searches (Doctor Google)

Practitioners reported a common trend of clients seeking antibiotics based on online recommendations or searches (e.g., Dr. Google). The knowledge sought from the internet has created an authoritative stance, allowing customers to purchase antibiotics without a prescription. The demand for antibiotics on the basis of online searches is common in urban areas.

“They come in pretending to know what exactly they want, but when you question them further, you realise it's because they have been reading online” (Pharmacist #3, Urban).

• Historical Patterns of Antibiotic Use

Clients also requested antibiotics based on successful past experiences with antibiotic use. Their ability to recall the specifics regarding the antibiotics was also reassuring to practitioners who dispensed the antibiotics without hesitation.

A practitioner highlighted his experience with a customer demanding an antibiotic: *“I have been taking this medicine before you were born, give it to me” (Pharmacist #5, Urban).*

• Brand Consciousness

Some clients exhibited strong preferences for particular antibiotic brands and described the brands as the only solution to their health crisis.

A patient brought a prescription for Artemether Lumefantrin and insisted that we add Augmentin since that is her usual go-to drug when she gets these symptoms. Upon further attempts to convince her otherwise, she insisted that she knows her body and that the doctor is not her usual doctor. I didn't fight it, I gave it to her – after all, I needed my money (Pharmacist #3, Urban).

## 5 Subjective norm

### 5.1 Themes

4 and 5 align with the “Subjective Norm” construct of the TPB, highlighting how perceived expectations from others, such as supervisors, customers and peers, influenced practitioners' willingness to dispense OTC antibiotics.

### 5.2 Theme

4: Competency Evaluation.

• Sales Targets

Some pharmacy owners have adopted the use of sales targets as a metric for employee assessment, which places practitioners in a difficult position to fully implement the requisite antibiotic control protocols. Although not all the pharmacies had defined sales targets, those without targets also felt that it was expected that they would produce good sales. The imposition of defined sales targets with corresponding incentives was more common in urban areas.

“It's a whole chain of events.” “As a pharmacist, if you are the proprietor of the facility, then you have the sole right to decide what to do, but when you are an employee, your employer expects you to meet certain sales targets” (Pharmacist #2, Urban).

• Positive Client Feedback

The practitioners described the willingness to supply antibiotics over the counter as a means of maintaining good client relationships, which earned the satisfaction of clients and sometimes resulted in gifts from customers.

I have one special customer from Nigeria, and I don't joke with him at all. When he comes, I need to find any medicine he wants. If I don't have it, I will look for it. ‘He treats me well’… she proceeds to say in the local Twi dialect (MCA #6, Peri-Urban).

• Effective Stock Management

The non-prescription supply of antibiotics in community pharmacies was also tied to a stock management procedure aimed at disposing of nearly expired products. Some antibiotics were considered slow-moving products, and any request, whether with a prescription or without, was welcome.

### 5.3 Theme

5: Social Networks.

• Customer Elitism/High Profile Clients

Suppliers felt more confident when the consumers demonstrated a relatively higher level of superiority or sophistication. This could be expressed in terms of higher education, wealth, or social status within the country.

“We determine why they need the antibiotics and if they merit it, we supply. The more educated a consumer is, the more confident I feel in supplying the antibiotic over the counter” (Urban Pharmacist #1).

If a whole Minister asks for an antibiotic, of course, he knows what he is asking for, and if you don't give it to him, he will, by all means, get it (MCA #6, Peri-Urban).

• Business Owner Associates

The social network of a person also informed the demand for antibiotics. Clients who were affiliated with the business owners expressed an inherent right to obtain prescription medicines, with little or no questioning from practitioners.

Ei Madam's friend! Please, they will go and report me, I don't want trouble (MCA #2, Urban).

• Medical Practitioner Associates

Most practitioners highlighted a common practice among clients, who referred to prior communication with a health worker as the basis for their antibiotic demands. The category of health worker affiliations differed slightly among the communities. The urban sector reported a standard reference to clients saying, “My friend is a doctor”, whereas the rural community referred to nurses as friends.

They request antibiotic IVs when you ask them why; most of the time, they will tell you that their sister or brother is a nurse. There are many nurses in this community, and most of them stay in compound houses. They are like the medical champions there” (MCA #4, Rural).

They like to throw around big names. A customer once said, “Do you know the head of cardio at Korle-Bu? He is my friend and has asked me to buy this antibiotic?” (Pharmacist, Urban #3).

## 6 Perceived behavioural control

### 6.1 Themes

6 and 7 correspond to the “perceived Behavioural Control” component of the TPB. They illustrate how the external environment shaped practitioners' perceptions of the ease or difficulty in supplying antibiotics over the counter.

### 6.2 Theme

6: Pandemic-Induced Dispensing.

• Psychosocial Emotions

The pharmacy practitioners reported greater ease in supplying antibiotics for COVID-19-related concerns. Panic, fear, media, lack of information, travel, anxiety, and a sense of vulnerability were associated with the demand for and supply of antibiotics during the pandemic.

• Lack of Information

Respondents communicated an increased distribution of antibiotics during the COVID-19 pandemic due to information scarcity. Due to the novelty of the situation and the absence of clear guidelines, practitioners had no qualms dispensing antibiotics over the counter.

“I was okay giving out azithromycin for COVID-19 because eventually that was what we were advised to give as professionals. (Pharm Tech #1, Peri-urban).

• Economic Survival

There was also a strong economic incentive to supply antibiotics at the peak of the pandemic. Businesses had taken a downward spiral, and it was not uncommon to adopt measures to boost sales. The respondents also reported an increased demand for more expensive brands during the pandemic, but this varied across different socioeconomic groupings. The respondents communicated that azithromycin was the most popular antibiotic of choice. However, members of highly urbanised/residential communities were more brand sensitive and preferred Zithromax only. Unlike the urban and peri-urban communities, the respondents in the rural sectors reported a relatively higher level of ‘customer naivety’ about the pandemic and the popular antibiotics of choice.

“Often, during the pandemic, a customer would walk in and say, ‘I have heard that there is an antibiotic that can be used for COVID-19 prevention, I want to buy it’. ‘They were asking for ‘Aziromyses' and ‘Erithromazin’; but actually, they were referring to azithromycin (Rural, MCA #4).

### 6.3 Theme

7: Weak Regulatory Controls.

• Ineffective Regulatory Oversight

Pharmacy practitioners described the regulatory system for antibiotics as generally weak. They indicated that a well-functioning regulatory framework was an incentive to uphold ethical standards. When quizzed about their personal responsibility to maintain professional standards, they responded that such choices are detrimental to the business, as if you do not supply, another shop will gladly satisfy your customer.

“I blame the same regulators because they make these products (antibiotics) available at wholesale to be issued to all outlets. I think the control systems are weak. Very weak! Professionals can prescribe within the standards, but if there is an outlet, whether a pharmacy or not, that can source antibiotics from the wholesale, then what's the point? (Pharmacist #4, Peri-Urban).

The last time I was surprised, I went to buy prescription medicines (including oral and IV antibiotics) from the wholesale stores at Okaishi – I am not usually the one who purchases our stock, so I was expecting the attendants to request some form of verification. To my shock, no one asked me anything.” (MCA #4, Rural).

• Business Proprietor Autocracy/Job Retention

Practitioners who were employees of the pharmacy expressed concern about the authority of business owners, which superseded the ethical expectations of the practice. Due to this situation, employee practitioners were under a sole expectation of driving sales rather than providing ethical pharmaceutical care. Hence, it was in the interest of the practitioners to align with the business principles as a means of maintaining their jobs.

## 7 Knowledge

### 7.1 Themes

8 and 9 represent the “Knowledge” component of the TPB. They capture practitioners' understanding of antibiotic use and AMR. This includes how their level of training, professional experience, and policy awareness shaped their confidence in dispensing antibiotics without a prescription.

### 7.2 Theme

8: Educational level of Supplier.

• Degree in Pharmacy

Practitioners with a degree or qualification in Pharmacy demonstrated a strong sense of awareness about antibiotics, the ‘prescription statuses of antibiotics and the concept of antimicrobial resistance (AMR). They presented the highest level of knowledge on antibiotics, but had no idea regarding the existence of a national antimicrobial policy or national antimicrobial plan. “*I have never heard of a National Antimicrobial Plan or Policy, what is that?” (Urban, Pharmacist #2).*

• Diploma/Short Course Training

The majority of the respondents were Medicine Counter Assistants (MCAs). MCAs were pharmacy practitioners who had undergone a 6-month training on the basics of pharmacy practice. Their training was intended to help pharmacists in the pharmacy. Yet most pharmacies, especially in the rural areas, were left under the sole control of the MCAs. They demonstrated the lowest knowledge on antibiotics/AMR and were more inclined to make sales.

### 7.3 Theme

9: Technical Expertise.

• Clinical Experience/Legal Mandate

Pharmacy practitioners expressed ease in the non-prescription dispensing of antibiotics based on their clinical expertise and their legal mandate as professionals. The clinical experience enabled them to assess risk-free choices in non-prescription antibiotic dispensing, which was well within their rights. This view was standard among pharmacists, and MCAs also indicated this perspective as long as they were acting in the stead of the pharmacist. The practitioners demonstrated knowledge about the prescription status of antibiotics, but maintained that it was within their purview to decide whether to supply antibiotics or not. Most practitioners restrict their non-prescription supply to the range of oral antibiotics, excluding intravenous (IV) dosage forms. However, a few others freely supplied IV antibiotics. These suppliers were primarily found in rural sectors.

“On the basis of my knowledge and expertise, I decide which antibiotics to give out. Yes, I understand that these are prescription medications, but they can also be prescribed by pharmacists based on our judgment. In the range of antibiotics mentioned earlier, I can prescribe them.” (Pharmacist #4, Peri-urban).

• AMR sensitisation

Prior AMR campaigns or engagements also informed practitioners' willingness to dispense antibiotics over the counter. The respondents highlighted AMR knowledge from lectures held as part of a Continuing Professional Development, In-house trainings, past academic lectures or as part of conferences.

## 8 Discussion

Various studies have substantiated the phenomenon of non-prescription supply of antibiotics in Africa, highlighting Over-the-Counter Medicine Counter Sellers (OTMCS) in rural areas as the principal drivers.^19^^,^^23^^,^^46^ Although OTMCS were not included in this study, similar patterns of over-the-counter antibiotic dispensing were observed in rural-based pharmacies. The non-prescription demand for antibiotics is notably common in this sector, and local jargons and colour codes have emerged as informal descriptors for specific antibiotics.^47^ Pharmacy practitioners have adopted these community-based interpretations, reinforcing a normalised view of casually dispensed antibiotics.^20^^,^^23^^,^^47^ This normalisation has fostered a strong positive attitude among suppliers and consumers regarding the non-prescription dispensing of antibiotics.^20^^,^^36^

In the urban communities, practitioners described their customers as learned, brand-conscious individuals who know what they want. Yet, despite these differences in customer profiles across the rural and urban settings, comparable trends of non-prescription antibiotic demand were observed. Donkor et al. suggest that the trend in urban settings may be linked to a strong association between higher education and the propensity to self-medicate.^22^ As individuals feel more confident in their understanding of their illness and treatment, they are more inclined to demand specific treatments. This was evident in the study results, where ‘online Google searches’ were frequently cited by practitioners as a common source of antibiotic requests. A typical situation of a sophisticated, educated patient and an inevitable reality of healthcare evolution.^48^^,^^46^ A study on ‘patient use of the internet for health information’ characterised the consumer display of health information as a ‘perceived threat’ to the practitioner, triggering defensive reactions from health workers.^49^ Similarly, pharmacy practitioners in peri-urban and rural areas reported feeling intimidated when consumers appeared to be knowledgeable or well-informed. In contrast, practitioners in urban settings communicated a sense of irritation towards such interactions, echoing sentiments shared by medical doctors who described the “internet-informed” patient as a hindrance to efficient time management.^46^^,^^50^ This dynamic reflects the impact of subjective norms, where the opinion of others exerts social pressure, influencing practitioners' responses in diverse ways.

‘Economic incentives from antibiotic sales’ and ‘customer retention’ were also recurring factors associated with practitioners' favourable views towards the non-prescription supply of antibiotics. The observation is consistent with findings from Vietnam, where both urban and rural pharmacy practitioners described the non-prescription sale of antibiotics as a means of business sustainability.^51^ In Africa, the high burden of infectious diseases creates a consistently favourable market for antibiotic sales, further reinforcing this practice.^52^^,^^53^

While economic incentives are an everyday basis for antibiotic supply, the perspectives of practitioners differ slightly across socioeconomic positions.

Across the study sites, the knowledge component of the Theory of Planned Behaviour (TPB) appeared to moderate dispensing behaviour. Pharmacy practitioners' understanding of antibiotic use and antimicrobial resistance (AMR) influenced how they rationalised their decisions. In rural and peri-urban areas, where pharmacy personnel were primarily MCAs, the non-prescription sale of antibiotics was more directly linked to financial gain.^23^^,^^54^ However, pharmacists, particularly in urban settings, described the issue as more complex and multifaceted, often framing it as an ethical challenge of balancing business interests with professional ethics. Bahnassi, (2015) reports a similar situation in Syria, where pharmacists struggled to balance moral obligations with market realities. The conundrum of the pharmacist in the business arena of community practice dates as far back as half a century.^55^, ^56^, ^57^ Doctors in private practice have similarly faced such crises, where factors such as financial incentives and the influence of Big Pharma compromise the clinical decision-making.^58^

In the context of this study, the ethical complexity faced by pharmacists is reinforced by strong subjective norms shaped by client expectations and pressure from business owners. Unfortunately, weak regulatory enforcement in Ghana further reinforces a perception of perceived behavioural control, creating an environment where the non-prescription supply of antibiotics is seen as both feasible and unlikely to result in sanction.^18^ When combined, these factors strengthen the behavioural intention to supply antibiotics over the counter. Interestingly, despite the differing explanatory pathways, most practitioners ultimately made the same decision to supply antibiotics without prescriptions. Non-pharmacist suppliers tended to be more transactional, often dispensing with minimal inquiry into antibiotic use. Pharmacists, on the other hand, were more likely to ask questions before supplying. However, these questions appeared to influence the choice of antibiotic rather than the decision to supply itself.

The COVID-19 pandemic equally modified the evolving landscape of non-prescription antibiotic use, heightening the impact of psychological factors such as fear and anxiety.^18^^,^^59^ During this period, attitudes and beliefs surrounding the pandemic strongly influenced suppliers' behavioural intentions to supply antibiotics over the counter. Despite awareness of antimicrobial resistance (AMR) and existing regulatory requirements, pharmacists still felt justified in dispensing OTC antibiotics.^18^^,^^60^

In Ghana, the Health Professions Regulatory Bodies Act 2013 (Act 857) serves as the overarching law that regulates the practice of pharmacies, which includes the sale, dispensing and purchase of medicines.^61^ Despite the explicit characterisation of an antibiotic as a prescription drug in Act 857, pharmacy practitioners exhibited no qualms about the non-prescription supply of antibiotics. Most pharmacists attributed this flexibility to a provision of the law that states, “A pharmacist or licenced pharmaceutical company may sell or supply prescription-only medicine to a person without a valid prescription if the supplier of the medicine reasonably believes that the person to whom the medicine is to be supplied is the proper person”.^61^ The ambiguity of this provision serves as a legislative basis, reinforcing the suppliers' perceived behavioural control, which warrants the supply of antibiotics without a prescription. This perceived legislative flexibility in antibiotic dispensing was clearly not exercised solely by pharmacists. Medicine counter assistants equally demonstrated broad discretionary powers in antibiotic supply, operating as independent prescribers in some facilities. In some cases, community pharmacies had evolved into ‘mini clinics’, where antibiotic injections were administered under the nominal oversight of MCAs. Such practices reflect a heightened sense of perceived behavioural control, where the lax regulatory environment empowers pharmacy practitioners to assume roles beyond their professional scope of work.

In Adikhari's paper on the motivations underlying over-the-counter purchase of antibiotics, Clinicians in South Asia described policies surrounding antibiotic use as ‘evadable policies’.^62^ This study revealed that practitioners demonstrated a total ignorance of the existence of a National Antimicrobial Policy and a National Antimicrobial Plan. Suggesting antibiotic-associated policies in Ghana can be described not only as ‘evadable policies’ but also as ‘ghost policies.’ This practitioner naivety is of concern, especially as antibiotic retailers constitute a core part of the strategy.^63^ It is noteworthy that the majority of participants were MCAs, and a gap in technical knowledge is anticipated.^64^ Nonetheless, given that these individuals were functioning in place of licensed pharmacists, they are expected to possess or demonstrate sufficient knowledge to ensure patient safety.^26^ MCAs have expressed interest in receiving formal training and recognition on antibiotic dispensing, but this has been met with strong opposition from policy actors.^65^

The collective insights from pharmacy practitioners across rural, urban, and peri-urban settings illustrate how attitudes, subjective norms, and perceived behavioural control interact with levels of community urbanisation to influence antibiotic dispensing practices in Ghana. In alignment with the TPB, the study demonstrates that non-prescription antibiotic supply is shaped not only by practitioners' own positive attitudes towards the practice, but also by external enabling factors such as weak regulatory enforcement, pandemic-induced anxiety and business-owner interests.^18^^,^^20^^,^^23^ These factors collectively influence a heightened sense of perceived behavioural control and reinforce the normalisation of non-prescription antibiotic dispensing. Although similar dispensing behaviours were observed across different practitioner groups, the underlying drivers varied significantly.

The study outcomes highlight the limitation of blanket strategies for behavioural modification on antibiotic dispensing and the need for context-specific targeted interventions.^26^^,^^65^^,^^66^ These insights are transferable to other LMICS that share comparable socio-cultural and professional dynamics, providing a behavioural framework to model community interventions on antibiotic control.

## 9 Implications for policy

Policy makers and regulators, such as the Ministry of Health, the Food and Drugs Authority (FDA), and the Pharmacy Council (PC), must take full cognisance of the factors outlined in this study and adopt community-tailored strategies that target both suppliers and consumers of antibiotics in Ghana.

Regulators of Pharmacy Education (Pharmacy Council) must ensure pharmacy training involves recognising patients/clients as full partners in healthcare decision-making and, as such, be eligible for a detailed explanation of rational antibiotic use. This also includes navigating away from situations of information asymmetry between pharmacy practitioners and clients.

Pharmacy practice regulators in Ghana, specifically the Pharmacy Council, may need to reinforce the authority of pharmacy practitioners to counter the prevailing normative belief that the opinion of pharmacy business owners supersedes the ethical obligation to control antibiotics.

The FDA and PC should ensure that continuous professional education on antibiotic control is provided for practitioners. The educational modules should be tailored to reflect practical field-based scenarios. It is necessary to recognise medicine counter assistants as an essential part of the local pharmaceutical supply chain in Ghana and, as such, adopt strategies that tend to advance their development.

Since current regulations allow for pharmacist discretion in antibiotic supply, it may be necessary for the FDA and PC to adopt a guidance framework for the over-the-counter supply of antibiotics in pharmacies to encourage rational antibiotic prescribing by pharmacists.

Finally, the Ministry of Health must ensure that essential policies, such as the National Antimicrobial Policy and the National Antimicrobial Plan, are widely disseminated among pharmacy practitioners to facilitate a more inclusive implementation process. This includes MCAs who appear to be an inevitable pillar of pharmacy practice in Ghana.

## 10 Limitations of the study

This study acknowledges some limitations worth noting. Firstly, as with most qualitative research, the study may be susceptible to the researchers' subjectivity. However, rigorous, structured, and comprehensive processes were employed during the study design, data collection and analysis to minimise a potential bias.^67^ Secondly, the findings are based on self-reported behaviours, which may be influenced by a social desirability bias. Due to the sensitivity of the subject, particularly in relation to professional ethics and knowledge, some respondents, especially pharmacists, may have responded in a manner that protects their image.^68^ However, ensuring data anonymity helped limit this by making participants more comfortable.^67^ Thirdly, despite efforts to recruit a diverse sample of pharmacy practitioners, the majority of participants were MCAs, which may have influenced the range of perspectives represented in the study.^69^ Furthermore, future research could employ mixed-method designs to assess the clinical consequences of OTC antibiotic use and explore how community trust and perceptions of pharmacists influence dispensing behaviour. Lastly, the study was conducted in a single region, which may limit the generalizability and transferability of the findings to other settings.

## 11 Conclusion

The degree of community urbanisation influences the scope of pharmacy practice and the modalities surrounding the non-prescription demand and supply of antibiotics in Ghana. Across these settings, the over-the-counter demand and supply of antibiotics in Ghana are fuelled by various factors, such as the influence of COVID-19, economic incentives, education, social networks, and the weak enforcement of antibiotic regulations. Notably, most pharmacies in Ghana, especially in rural and peri-urban settings, are primarily manned by medicine counter assistants with minimal experience and a significant sales-oriented positioning on antibiotic dispensing.

## CRediT authorship contribution statement

**Radolf Ansbert Nortey:** Methodology, Formal analysis, Data curation, Conceptualization, Writing – review & editing, Writing – original draft. **Irene Akwo Kretchy:** Supervision, Formal analysis, Conceptualization, Writing – review & editing. **Mercy Naa Aduele Opare-Addo:** Supervision, Project administration, Conceptualization.

## Consent for publication

Not applicable.

## Ethics approval and consent to participate

The study was approved by the Ghana Health Service Ethics Committee on 11th October 2021 under the registration code GHS-ERC: 008/05/21. The participants were allowed to consent to their involvement in the study after being made aware of the objectives. Participation was based on willingness and responses were anonymous.

## Funding

No funding was received for conducting this study.

## Declaration of competing interest

The authors declare that they have no known competing financial interests or personal relationships that could have appeared to influence the work reported in this paper.

## Data Availability

Most of the anonymised data have been included in this manuscript, and providing individual transcripts will compromise the confidentiality and anonymity criteria of the ethical approval process. All interested researchers who meet the criteria for access to confidential data with appropriate ethical approval can, however, access the data set from the corresponding author via the email address ansbertn@gmail.com
